# Induction of double-strand breaks with the non-steroidal androgen receptor ligand flutamide in patients on androgen suppression: a study protocol for a randomized, double-blind prospective trial

**DOI:** 10.1186/s13063-023-07838-4

**Published:** 2023-12-16

**Authors:** Emerson Lee, Jonathan Coulter, Alok Mishra, Fernanda Caramella-Pereira, Angelo Demarzo, Michelle Rudek, Chen Hu, Misop Han, Theodore L. DeWeese, Srinivasan Yegnasubramanian, Daniel Y. Song

**Affiliations:** 1 Johns Hopkins University School of Medicine, Baltimore, USA; 2 Department of Urology, Johns Hopkins University, Baltimore, USA; 3 Department of Oncology, Johns Hopkins University, Baltimore, USA; 4 Oncology Pathology, Johns Hopkins University, Baltimore, USA; 5 Department of Biostatistics, Johns Hopkins University, Baltimore, USA; 6 Department of Radiation Oncology and Molecular Radiation Sciences, Johns Hopkins University, Baltimore, USA

## Abstract

**Background:**

Prostate cancer remains the most prevalent malignancy and the second-leading cause of cancer-related death in men in the USA. Radiation therapy, typically with androgen suppression, remains a mainstay in the treatment of intermediate- and high-risk, potentially lethal prostate cancers. However, local recurrence and treatment failure remain common. Basic and translational research has determined the potential for using androgen receptor (AR) ligands (e.g., dihydrotestosterone and flutamide) in the context of androgen-deprived prostate cancer to induce AR- and TOP2B-mediated DNA double-strand breaks (DSBs) and thereby synergistically enhance the effect of radiation therapy (RT). The primary aim of this study is to carry out pharmacodynamic translation of these findings to humans.

**Methods:**

Patients with newly diagnosed, biopsy-confirmed localized prostatic adenocarcinoma will be recruited. Flutamide, an oral non-steroidal androgen receptor ligand, will be administered orally 6–12 h prior to prostate biopsy (performed under anesthesia prior to brachytherapy seed implantation). Key study parameters will include the assessment of DNA double-strand breaks by γH2A.x foci and AR localization to the nucleus. The initial 6 patients will be treated in a single-arm run-in phase to assess futility by establishing whether at least 2 subjects from this group develop γH2A.x foci in prostate cancer cells. If this criterion is met, the study will advance to a two-arm, randomized controlled phase in which 24 participants will be randomized 2:1 to either flutamide intervention or placebo standard-of-care (with all patients receiving definitive brachytherapy). The key pharmacodynamic endpoint will be to assess whether the extent of γH2A.x foci (proportion of cancer cells positive and number of foci per cancer cell) is greater in patients receiving flutamide versus placebo. Secondary outcomes of this study include an optional, exploratory analysis that will (a) describe cancer-specific methylation patterns of cell-free DNA in plasma and urine and (b) assess the utility of serum and urine samples as a DNA-based biomarker for tracking therapeutic response.

**Discussion:**

This study will confirm in humans the pharmacodynamic effect of AR ligands to induce transient double-strand breaks when administered in the context of androgen deprivation as a novel therapy for prostate cancer. The findings of this study will permit the development of a larger trial evaluating flutamide pulsed-dose sequencing in association with fractionated external beam RT (+/− brachytherapy). The study is ongoing, and preliminary data collection and recruitment are underway; analysis has yet to be performed.

**Trial registration:**

ClinicalTrials.gov NCT03507608. Prospectively registered on 25 April 2018.

## Background

Although active surveillance for very low-risk prostate cancer (PCa) has become prevalent, evidence for the importance of local therapy for patients with intermediate- and high-risk and even oligometastatic disease is growing [1, 2]. The pivotal role of androgen signaling in prostate cancer progression has long been recognized, and suppression of androgen receptor-mediated effects on prostate cancer remains a pillar of its clinical management. For high-risk, potentially lethal prostate cancers, radiation is a local treatment modality with level 1 evidence demonstrating improved survival when administered in concert with androgen suppression [2, 3]. However, despite the value of this combined approach, prostate cancer remains the second leading cause of cancer-related mortality in men in the USA [4, 5]. Local recurrence remains the predominant mode of treatment failure, and strategies to improve the therapeutic index of radiation-based therapy of intermediate- and high-risk prostate cancer are critically needed [3].

We and others have previously shown that stimulating AR transcriptional programs in androgen-deprived prostate cancer cells using natural AR agonists such as testosterone and dihydrotestosterone (DHT) results in the production of numerous, transient DNA double-strand breaks (DSBs) that require DNA repair machinery for resolution [6–9]. These double-strand breaks are mediated by the type II topoisomerase TOP2B, which essentially acts as a transcriptional co-activator with the AR. Although the mechanistic causes and endogenous role of these DSBs in AR signaling are unclear and are the subject of ongoing research, the finding that AR stimulation could lead to TOP2B-mediated DSBs was highly reproducible and robust and presented a potential novel paradigm of exploiting AR signaling to sensitize prostate cancer cells to other therapeutics [8, 10]. In preclinical studies, the DSBs produced by treatment of cells with a pulse of DHT led to significant selective sensitization of AR-positive cells to treatment with ionizing radiation both in vitro and in vivo [8]*.*

However, the prospect of overt AR stimulation, even transiently, in the treatment of localized prostate cancer poses difficulties in clinical implementation given the dependence on and stimulation of prostate cancer growth from AR signaling. We hypothesized that other AR ligands, including some of the current non-steroidal AR antagonists, may similarly be able to partially stimulate the AR and selectively induce TOP2B-mediated DSBs without concurrent activation of AR-mediated growth stimulation and transcriptional programs. Among a series of tested AR antagonists, hydroxyflutamide (the major active metabolite of flutamide) induced nuclear translocation of AR and stimulated TOP2B-mediated DSBs at levels comparable to saturating levels of the AR agonist DHT [11]. RNAi-mediated knockdown of either AR- or topoisomerase 2 beta (TOP2B) prevented DSBs resulting from hydroxyflutamide (HF) exposure [11], which is consistent with mechanistic observations following stimulation with DHT. This was not simply due to the presence of AR mutations that induce an antagonist to agonist switch (e.g., in AR T877A mutation LNCaP cells), since cell lines without such mutations (LAPC4, VCaP, CWR22RV1) also showed similar formation of DSB with HF [11]. The combination of HF with ionizing radiation synergistically induced double-strand breaks and led to the inhibition of clonogenic survival compared to either agent alone. Stimulation of castrated nude mice implanted with prostate cancer xenografts with a pulse of HF followed by treatment with ionizing radiation also led to significant growth inhibition of the xenografts compared to mice treated with HF alone or ionizing radiation alone, suggesting the therapeutic benefit of the paradigm in vivo (Unpublished data, personal communication from JC, TLD, SY).

In summary, this work suggests that TOP2B-mediated DNA DSBs and radiosensitization elicited by AR ligands such as HF could have clinical utility when utilized in a pulse-dose fashion with fractionated radiotherapy. If also confirmed in humans, this novel paradigm may be readily translatable to clinical practice. Such an effect would enhance the therapeutic index of radiotherapy given its high specificity to AR-expressing tissues and could be exploited as an adjunct to RT (either conventionally fractionated or hypofractionated) by dosing HF at spaced intervals over the course of treatment. *The primary goal of this study is to carry out a pharmacodynamic clinical trial to test whether a pulse of HF can induce DSBs in men undergoing androgen suppression as part of their treatment strategy for localized prostate cancer.*

### Objectives

#### Primary objective

To confirm that DNA double-strand breaks occur in prostate cancer tissue following administration of pulse-dose flutamide administration in androgen-suppressed patients, as compared to control patients receiving placebo.

#### Exploratory objectives

Aim 1: To explore the sensitivity of detecting cancer-specific alterations in DNA methylation at a panel of frequently hypermethylated loci (e.g., GSTP1, PTGS2, MDR1, APC, RARbeta, gene promoters) within urine and plasma.

Aim 2: To examine whether prostate cancer-specific DNA methylation alterations can be used as a DNA-based biomarker for tracking therapeutic response.

Aim 3: To explore the kinetics for these measures when compared with PSA values and clinical response.

### Study population

Patients with a diagnosis of adenocarcinoma of the prostate will be recruited and treated in the Department of Radiation Oncology at our single academic medical center. Patients consulted are deemed eligible for brachytherapy based on having histologically confirmed, localized (M0) adenocarcinoma of the prostate (not post-prostatectomy), gland size ≤ 50 cc, absence of severe lower urinary tract obstructive symptoms (International Prostate Symptom Score ≤ 15), and no prior transurethral resection of the prostate (TURP). Of this subset, approximately two-thirds will receive androgen suppression with brachytherapy due to intermediate- or high-risk disease. Minorities of all races and ethnic groups are eligible for this study and will be encouraged to participate. Based on demographic trends in our center, we project that 24% of men in this study will be non-white.

Additional inclusion criteria include the following:

- Clinical stages T1c–T3b, Mx or M0
- At least one biopsy core with intermediate- or high-risk (Gleason 7 or higher) disease
- The patient has decided to undergo brachytherapy plus androgen suppression for his prostate cancer (with or without supplemental external beam radiation)
- Suitable volume of disease for biopsy, defined as one or more of the following:
  - MRI scan of the pelvis with ≥ 1 identifiable PI-RADS 4 or 5 lesion measuring at least 0.5cm in any dimension OR
  - Clinically palpable disease corresponding to (ipsilateral to) any involved core on biopsy
- All participants will sign an informed consent describing the objectives of the study and potential risks.

Biopsy and brachytherapy will be performed anytime within 180 days from initiation of androgen suppression using gonadotropin-releasing hormone (GnRH) agonist or antagonist, provided the patient’s testosterone level is < 50 ng/dl following androgen suppression.

Patients are ineligible if they have any of the following exclusion criteria:

- Known hypersensitivity or allergic response to flutamide
- Severe hepatic impairment (serum ALT level >2x normal or serum AST level >2x normal)
- Pre-existing diagnosis of hypogonadism (serum total testosterone < 150 ng/dL) prior to starting hormone therapy for prostate cancer, or treatment with testosterone supplementation therapy within 12 months prior to enrollment
- Major medical or psychiatric illness which, in the investigator’s opinion, would hinder or prevent completion of treatment

### Sample size justification and accrual

This trial will be utilized to design a larger study to establish measures for such parameters with a reasonable level of precision. We will enroll 30 patients in the trial, with a goal of at least 24 evaluable patients accrued.

In the run-in phase, a total of 6 evaluable patients will be accrued to receive Flutamide administration. We will use a Bayesian monitoring rule to determine if the study is feasible. Specifically, we would consider it “infeasible” if the probability of not observing greater than or equal to 5% of prostate cancer cells having γH2Ax foci is more than 50%. Based on the Bayesian monitoring rule in Section 6.3, if there is less than or equal to 1 out of 6 cases with detectable DNA double-strand breaks (i.e., greater than or equal to 5% of prostate cancer cells having γH2Ax foci on patient level), the study will be considered as infeasible and will not proceed into the randomization part.

If the study proceeds into the randomization part, a total of 18 evaluable patients will be randomized in a 2:1 ratio to either receive flutamide (12 patients) or not (6 patients). A simple randomization method will be utilized. A total of 18 patients treated with flutamide (6 from run-in and 12 from randomization) will produce a two-sided 90% confidence interval with a width ranging from 0.35 to 0.42 (if the true proportion of prostate cancer cells having γH2Ax foci is 30%-80%). A total of 6 patients in control will produce a two-sided 90% confidence interval with a width approximately less than 0.5 (if the true proportion of prostate cancer cells having γH2Ax foci is less than 10%).

All eligible patients will be offered enrollment in the study, and we estimate a 20% dropout and non-evaluable rate. Patients who drop out prior to the biopsy/brachytherapy procedure will be replaced; patients who drop out after the biopsy/brachytherapy will not be replaced. In order to ensure at least 24 evaluable patients are available, we plan to enroll ~30 patients in this trial. Given the minimal addition to standard therapy and effort requirement from patients, we expect to accrue at least 2–3 patients/month, and the full accrual will be completed in 2 years.

The feasibility of detecting DNA double-strand breaks will be based on the proportion of patients in whom γH2Ax foci are observed in ≥5% of prostate cancer cells. Conversely, the trial will be considered “infeasible” if the probability of failure to observe DNA double-strand breaks is more than 50% and with more than 70% posterior probability. We assume there will only be a smaller proportion of cases in which DNA double-strand breaks cannot be observed, e.g., on average only 10% of patients will not have detectable DNA double-strand breaks and there will be about 16% chance that the risk will be 20% or more. This corresponds to a Beta (0.1, 0.9) prior distribution. Table 1 summarizes the continuous termination rule for the 18 evaluable patients treated with flutamide. The feasibility termination rule calls for the conclusion of the study without proceeding into the randomization part if there is less than or equal to 1 patient with detectable DNA double-strand breaks within the first 6 patients (in the run-in phase). If the trial enters the randomization phase, the feasibility-stopping rule calls for accrual suspension if the number of patients with detectable DNA double-strand breaks is too low.

**Table 1:** Stopping rule for feasibility

# patients without detectable DNA double-strand breaks	5	6	7	8	9	10	11
Out of the total # evaluable patients	6–7	8–9	11	12–13	14–15	16–17	18

Table 2 summarizes the operating characteristics based on 5000 simulations with 18 evaluable patients in terms of how frequently the study would stop based on the stopping rule under different hypothetical feasibility rates, as well as the average sample sizes.

**Table 2:** Operating characteristics of the stopping rule for feasibility

Underlying proportion without detectable DNA double-strand breaks	0.4	0.5	0.6	0.7	0.8
% of the time study stops	19.3%	44%	74.8%	92.4%	99.4%
Expected sample size	16.3	14.1	11.1	8.7	6.9

## Methods/design

This will be a prospective, single-center, two-phase pilot study to preliminarily evaluate if DNA double-strand breaks occur in prostate cancer tissue following pulse-dose flutamide administration within patients receiving central androgen suppression and brachytherapy. It consists of two phases, a run-in phase and a randomized phase. The run-in cohort of 6 patients is for preliminary determination of feasibility and exclusion of futility, i.e., to confirm that DNA double-strand breaks (γH2Ax foci) are observed. Assuming non-futility in the run-in phase, the accrual of participants for the randomized phase will take place. In this randomized, double-blind phase, subjects will be randomized in a 2:1 ratio (12:6 patients), for a total of 12 patients randomized receiving flutamide and 6 patients receiving placebo. This phase aims to confirm the flutamide treatment effect by comparing proportions of prostate cancer cells having γH2Ax foci and the distribution of number of foci in cancer cells in patients treated with flutamide versus patients receiving a placebo.

**Fig. 1:**
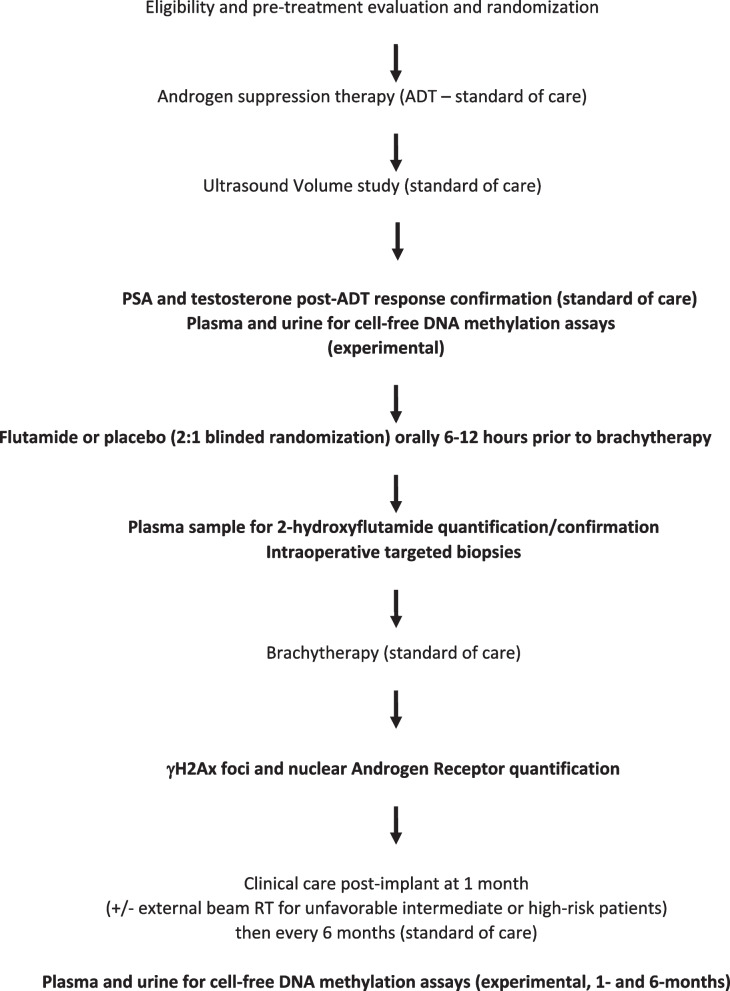
Trial Protocol Schema denoting research vs standard of care interventions

**Fig. 2.**
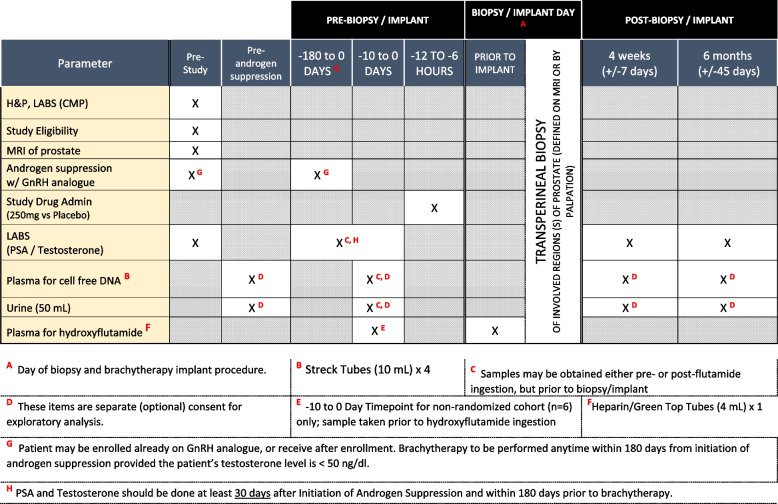
SPIRIT figure [12], clinical assessments, and study calendar

Patients enrolled in both phases will receive standard-of-care treatment with the addition of (a) pre-procedure flutamide or placebo, (b) intraoperative transperineal biopsy prior to brachytherapy seed implantation, and (c) optional experimental correlative assays. In brief, following patient evaluation and determination of trial eligibility, initial patients will be allocated to the run-in treatment group (run-in phase), or if the run-in phase is completed with confirmatory results, then subsequent patients will be randomized to either the treatment group or placebo (randomized phase). They will undergo routine pre-procedure laboratory studies (comprehensive metabolic profile, PSA, testosterone), MRI of the prostate, and initiation of androgen suppression with GnRH analog. In patients consenting to the secondary experimental studies, plasma and urine samples will be collected according to the schedule delineated below. Six to 12 h prior to the procedure, patients will take either 250 mg flutamide or placebo. Our on-site research pharmacy service will repackage both flutamide and placebo doses in identical generic capsule form, thereby blinding patients to their treatment assignment. Clinical and laboratory investigators will also be blinded to patient assignment, with the treatment arm only known to research trial staff. Intraoperatively, all patients will undergo MRI-guided, cognitive-fusion transperineal biopsy immediately prior to interstitial seed implantation. Postoperative care will be standard. Figure 1 depicts the trial flowchart, and Fig. 2 denotes the timeline of standard-of-care, trial, and experimental studies. Patients with unfavorable intermediate- or high-risk disease may also receive external beam radiotherapy in addition to brachytherapy, consistent with consensus guidelines.

### Plasma assay for 2-hydroxyflutamide

2-Hydroxyflutamide levels will be assayed both pre- and post-flutamide dosing in the initial non-randomized cohort. For the randomized cohort, only post-flutamide levels of 2-hydroxyflutamide will be assayed.

Blood samples (~4 ml) will be drawn into heparinized test tubes 0–10 days before drug administration (non-randomized initial cohort only), and also at 6–12 h post-ingestion of flutamide. Blood samples are to be immediately placed on ice after collection and centrifuged at 1300*g* for 10 min at 4 °C, and the plasma fraction is to be separated and stored in polypropylene tubes at temp of −70 °C until analysis.

For analysis, specimens will be transferred to the Analytical Pharmacology Core Laboratory and analyzed based on a method described by Niopa et al.

### Biopsy and implant procedure

In the operating room, patients will be anesthetized per clinical routine for brachytherapy and placed in a dorsal lithotomy position. A transrectal ultrasound (TRUS) probe will be placed into the rectum for prostate visualization. The perineum will be prepped in a sterile fashion.

TRUS images will be captured and fused with preoperative MRI to identify target lesions. Transperineal biopsy will be carried out under TRUS guidance, with ≥3 cores targeted per MRI-defined region of involvement and/or clinically palpable region. The number of biopsy cores taken is not to exceed twelve cores. Following the completion of the biopsy, patients will undergo brachytherapy per usual clinical routine.

Biopsy samples will be processed for paraffin embedding and pathologic confirmation of tumor tissue presence. Analyses for DSBs will be performed using assays for γH2Ax, and secondarily for other markers such as TUNEL and 53BP1. Approximately 1/3 of the tissue will be frozen and set aside for future laser capture microdissection for RNA sequencing.

Central pathology review will be performed for patients who have agreed/opted to allow us access to any post-treatment biopsies, if performed in routine clinical care, and retrospective review of baseline diagnostic pathologies. DNA extracted from formalin-fixed biopsy tissue (using the Qiagen FFPE DNA isolation kit) may be used for correlative DNA methylation analysis and will be analyzed using the same methods for COMPARE-MS and MBD-seq described below.

### DNA damage in biopsy tissue

Biopsy tissues will be fixed in 10% buffered formalin and subjected to paraffin embedding (JHH Pathology) and pathologic review of hematoxylin and eosin-stained sections. Immunostaining will be performed manually and images will be scanned with a ×40 objecting using Ventana DP200 (Roche Diagnostics) digital whole slide scanning system. γH2Ax (Cell Signaling) and AR (Millipore-Sigma) staining will be performed and visualized using the 3-amino-9-ethylcarbazole (AEC) chromogen. Following initial γH2Ax staining, antibody stripping will be performed using 95% ethanol washes until no visible AEC product remains as previously described [13] prior to AR immunostaining of the same section. Nuclear γH2Ax foci and total nuclear AR signal intensity colocalizing with hematoxylin will be quantified using HALO 3.6 (Indica Labs) software by a urologic pathologist blinded to the sample groups and reported as γH2Ax foci per cell and nuclear AR, respectively.

### Plasma, urine, and serum sampling for analyses

#### Plasma assay for 2-hydroxyflutamide

2-Hydroxyflutamide levels will be assayed both pre- and post-flutamide dosing in the initial non-randomized cohort. For the randomized cohort, only post-flutamide levels of 2-hydroxyflutamide will be assayed.

Blood samples (~4 ml) will be drawn into heparinized test tubes 0–10 days before drug administration (non-randomized initial cohort only), and also at 6–12 h post-ingestion of flutamide. Blood samples are to be immediately placed on ice after collection and centrifuged at 1300*g* for 10 min at 4 °C, with separation of the plasma fraction and storage in polypropylene tubes at −70 °C until analysis. Specimens will be transferred to our Analytical Pharmacology Core Laboratory and analyzed based on the method described by Niopas et al. [14].

#### Plasma and urine exploratory analyses

As an exploratory analysis exploring the feasibility of detecting cancer-specific alterations (including DNA methylation patterns at frequently hypermethylated loci, e.g., GSTP1, PTGS2, MDR1, APC, RARbeta, gene promoters) and their utility as a DNA-based biomarker for tracking therapeutic response, serum and urine samples will be collected and analyzed for methylation alterations in a specific panel of genes using COMPARE-MS [15, 16] and/or in a genome-wide fashion using MBD-seq [17–21]. Samples will be collected at 4 time points: prior to androgen suppression, after initiation of androgen suppression, 1 month post-implant, and 6 months post-implant. Patients will sign consent specifically for these samples or may choose to opt out. Given that these analyses do not affect the primary endpoint, patients opting out of the exploratory serum and urine analyses will not adversely affect the study endpoint. Additionally, a participant’s care will not be altered if they choose to opt out of the exploratory plasma/urine analysis portion of the study. As a part of the exploratory analysis consent, patients will also be consented to access to subsequent biopsy and corresponding PSA values that may be obtained through standard clinical care.

##### Blood collection

In most cases, blood samples will be drawn from patients scheduled for venipuncture for routine clinical purposes. When not possible, blood draws will occur at times other than those needed for routine clinical care. Generally, blood draws for research purposes will be no greater than necessary to fill 4 × 10mL tubes.

##### Blood processing

Blood will be drawn into 4 Streck tubes for collection and stored as plasma, serum, white blood cells, or whole blood. Serum will be separated from other cellular components by centrifugation, allocated into tubes, catalogued, and frozen at −70 to −80 °C or viably in liquid nitrogen freezers. Samples may be processed for DNA, RNA, and/or protein.

##### Urine collection and processing

Urine collected from patients is known to contain nucleic acid material that could serve as a biomarker for cancer. In addition, urine studies may involve the purification of proteins and/or cells. Urine preservative (Norgen BioTEK Corp, Cat # 18124) will be added to the urine and stored at room temperature or 4 °C until further processing.

##### Purification of nucleic acid from biospecimens (urine/plasma)

DNA purification from plasma and urine will be accomplished using a commercially available Qiagen virus vacuum kit (QiaVirus vacuum kit, Cat # 57714), starting with 1 ml of plasma or 10 ml of urine prepared as detailed above (thawed @ RT). The procedure for purification of plasma is as follows. In brief, we will transfer 500-μl plasma to two separate 2-ml tubes, add 40 μl Proteinase K and mix by vortexing, and add 5.6 μl 1μg/μl RNA carrier to 500 μl and mix by vortexing. The specimen will be incubated at 1 h @ 60 °C in a heating block, then cooled on ice before adding 600 μl EtOH, mixed thoroughly by vortexing, and incubated 5 min at room temperature. The sample (lysate) will be transferred to a vacuum purification column w/extension tube placed on a vacuum manifold. The column will be washed to purify the nucleic acids before the eluate is drawn off the column in nuclease-free molecular biology grade water (~100ul).

*COMPARE-MS assay for assessing DNA methylation at a panel of gene loci:* We will use the COMPARE-MS assay to assess the extent of DNA methylation in urine DNA and cell-free DNA from plasma at gene loci that are known to be commonly methylated selectively in prostate cancer cells. COMPARE-MS is a bisulfite-free method of DNA methylation analysis and will be performed as described in detail previously [16]. Briefly, DNA samples will be digested with AluI and HhaI restriction enzymes (New England Biolabs, Ipswich, MA), and methylated DNA fragments will be enriched using recombinant MBD2-MBD (Clontech, Mountain View, CA) immobilized on magnetic Tylon beads (Clontech). The enriched methylated DNA will be eluted and subjected to a real-time polymerase chain reaction (qPCR), with primers specific to sequences known to be selectively methylated in prostate cancer tissues, including regulatory sequences of the GSTP1, APC, PTGS2, ABCB1, and RARBeta genes. For quantitative assessment, we will use a standard curve composed of a dilution series of positive control M.SssI-mediated fully methylated male genomic DNA.

*MBD-seq for combined assessment of methylation alterations and structural variants genome-wide:* The MBD-seq approach involves the division of DNA extracted from each plasma and urine sample into two fractions: a total input fraction, from which structural alterations can be measured, and a methyl-binding domain (MBD)-enriched methylated fraction, from which DNA methylation changes can be measured. Briefly, each DNA sample from urine or plasma will be spiked in with a fully methylated lambda-phage DNA internal control, fragmented, and ligated to barcoded sequencing adaptors. Half the sample will be set aside as the total input, and the other half will be subjected to enrichment of methylated DNA using MBD-conjugated magnetic beads as we have described previously [15–18]. The resulting enriched methylated fraction and the total input fraction will then be amplified, and the resulting libraries will be subjected to paired end next-generation sequencing. In each fraction, the number of reads mapping to each of ~100 regions that we have previously shown to be highly recurrently (>50% of prostate cancers) and stably (maintained through the disease course and across metastatic dissemination) methylated in a prostate cancer-specific manner will be determined. The number of reads mapping to the fully methylated spiked-in internal quantitation standard will also be determined. The MI score will be defined as follows: MI = (H_E_/H_T_)/(S_E_/S_T_), where H_E_ and H_T_ are the number of reads mapping to the prostate cancer-specific hypermethylated regions in the enriched and total input fractions respectively, and S_E_ and S_T_ are the number of reads mapping to the spiked-in internal standard in the enriched and total input fractions respectively. Furthermore, the number of paired-end reads showing evidence of rearrangements (each sequence from a paired-end fragment mapping to discontiguous portions of the genome), determined as described previously [16, 19], in the total input fraction normalized per million overall reads (SR score) will provide a parallel measure of the amount of DNA containing genomic rearrangements. By measuring these alterations using an unbiased genome‐wide approach, we can be less limited by stochastic factors that can prevent assessment of a single locus. Furthermore, by measuring both the DNA methylation and structural alterations, we are less prone to technical issues that may limit measurement of any one of these alteration classes. Finally, assessment of both urine and plasma, both of which can be readily and non‐invasively obtained, will allow a higher chance of detecting these alterations. The accuracy and precision of each type of analysis will be explored using prostate cancer cell line DNA spiked in a dilution series into normal plasma and urine. 

Technical optimization of these exploratory methods may be needed in order to optimally work with real-world plasma and urine samples. 

### Feasibility of detecting cancer-specific DNA methylation alterations

Calculated MI score is related to the amount of methylated DNA derived from the top 100 regions that we have previously identified to be highly frequently and stably hypermethylated in human prostate cancer but not in any normal tissues assessed (including normal prostate, lymph nodes, spleen, liver, kidney, and blood). This approach essentially allows massively parallel assessment of all of these regions in a single assay. If in our exploratory analyses, we determine the need to include other regions or subtract regions, this can simply be done informatically without any changes needed to the actual assay. Furthermore, the number of paired-end reads showing evidence of rearrangements (each sequence from a paired-end fragment mapping to discontiguous portions of the genome), determined as described previously [17, 20], in the total input fraction normalized per million overall reads (SR score) will provide a parallel measure of the amount of DNA containing genomic rearrangements. By measuring these alterations using an unbiased genome-wide approach, we can be less limited by stochastic factors that can prevent the assessment of a single locus. Furthermore, by measuring both the DNA methylation and structural alterations, we are less prone to technical issues that may limit the measurement of any one of these alteration classes. Finally, assessment of both urine and plasma, both of which can be readily and non-invasively obtained, will allow a higher chance of detecting these alterations. The accuracy and precision of each type of analysis will be explored using prostate cancer cell line DNA spiked in a dilution series into normal plasma and urine.

### Follow-up evaluation

Patients will be assessed for adverse events per routine clinical practice at 1 month and 6 months post-implant, at which time serum and urine specimens will be collected for exploratory analyses. Given that prostate biopsies are routine clinical practice and also since flutamide is a drug already approved for clinical long-term use, the risk for toxicity related to participation in this trial is minimal. Patients will be considered off-trial following the 6-month follow-up visit.

### Reporting of serious or unexpected adverse events

There are no expected serious adverse events associated with this trial; flutamide is currently FDA-approved for the indication of prostate cancer. The descriptions and grading scales found in the revised NCI Common Terminology Criteria for Adverse Events (CTCAE) version 4.0 will be utilized for all AE reporting.

### Adverse event reporting

If a patient experiences an adverse event while on study, the following steps will be taken:

1. Establish the cause and severity of the adverse event and determine if said event is related to study participation.
2. Principal Investigator will decide what treatment(s), if any, is/are required.
3. Depending on the type and severity of an adverse event, an appropriate follow-up schedule will be constructed which will allow for determination of event outcome.
4. Patient will be followed up by the Principal Investigator until the adverse event has been resolved.

### Departure from the protocol

If there is a departure from the Clinical Protocol, the Principal Investigator will notify in writing both the local IRB at Johns Hopkins and the HSRRB at the time of annual review (continuing review). The research coordinator will keep a log of all deviations/departures that occur on this project and this log will be reviewed by the research team on a monthly basis. During the review, the research team will discuss corrective action plans to minimize future deviations/departures. If there are departures from the protocol that affect patient safety, the Principal Investigator will notify in writing the IRB within 24 h of discovering the departure/deviation. Once a patient is “off study,” their treatment and follow-up plan will conform to what is determined to be in their best interest at that point, and no longer according to the protocol.

### Roles and responsibilities of study personnel

*Principal Investigator*: Oversees all aspects of the trial. Recruits and consents patients and administrates protocol-specific procedures. Provides medical care to research subjects during the conduct of the study. Follows and advises regarding the treatment of adverse events. Reports SAEs to the JHM-IRB within the required time frame. Amends the trial as necessary to reflect unforeseen adverse events, new scientific data, and the general integrity of the study. Monitors the trial and is ultimately responsible for the conduct of protocol.

*Co-Investigators*: If a physician: can recruit and consent patients and can administrate protocol-specific procedures. Can provide medical care to research subjects during the conduct of the study. Has input on the course of action for adverse events.

If not a physician: collaborates with the Principal Investigator according to area of expertise.

Research Nurses: Can consent patients. Execute protocol-specific procedures requiring nursing qualifications. Provide nursing care to research subjects during the conduct of the study.

Data Manager/Study Coordinator: Collects data from subject’s medical records and codes it onto the study's case report forms. Notifies Principal Investigator of any deviations that he/she finds while managing the data. Prepares annual IRB renewals and termination report upon study completion, assists with the management of regulatory issues governing the trial. Monitors the trial.

### Ethical and regulatory considerations

IRB: Prior to initiating the study, the Principal Investigator must obtain written approval to conduct the study from the appropriate IRB. Should changes to the study protocol become necessary, protocol amendment will be submitted in writing to the IRB by the Principal Investigator for IRB approval prior to implementation.

Informed consent: All potential candidates for the study will be given a copy to read of the informed consent for the study. The investigator will explain all aspects of the study in lay language and answer all the candidate’s questions regarding the study. If the candidate desires to participate in the study, he/she will be asked to sign the informed consent. No study procedures will be performed on a patient until after they have signed the informed consent document. Subjects who refuse to participate or who withdraw from the study will be treated without prejudice.

The principal investigator will ensure that the study is conducted in compliance with the protocol and according to ICH Guidelines for Good Clinical Practices, the Declaration of Helsinki, and all regulatory and institutional requirements, including those for patient privacy, informed consent, Institutional Review Board approval, and record retention.

### Data analysis and statistical considerations

Following the conclusion of data collection for the run-in phase, the feasibility of the study will be evaluated as outlined above. We would consider the study “infeasible” if the probability of observing γH2Ax foci in ≥ 5% of prostate cancer cells is less than 50%. Based on the Bayesian monitoring rule described above, if one or fewer patients of the 6 patients accrued to the run-in phase of the study have observable γH2Ax foci at an appreciable level as delineated above, the study will be considered infeasible and will not proceed to randomization. Statistical analysis will be performed using R software (The R Foundation, Vienna, Austria).

The measurement of Flutamide may be accordingly transformed to ensure the necessary distributional and model assumptions are met. Analyses for all secondary endpoints will be exploratory in nature and specified in detail in the study protocol and statistical analysis plans.

### Internal data monitoring plan

This is a DSMP Level I study under the SKCCC Data Safety Monitoring Plan (12/6/2012). The Clinical Research Office QA Group will perform an audit after the first subject has been treated and then periodically depending on the rate of accrual and prior audit results. All trial monitoring and reporting will be reviewed annually by the SKCCC Safety Monitoring Committee. The PI is responsible for internally monitoring the study. Data must be reviewed to assure both the validity of data and the safety of the subjects. The PI will also monitor the progress of the trial, review safety reports, and clinical trial efficacy endpoints and to confirm that the safety outcomes favor continuation of the study.

Clinical records for all subjects studied including history and physical findings, laboratory and clinical data, and operative and dosimetric records are to be maintained by the investigators in a secure location at Johns Hopkins Cancer Center. Any records that are stored electronically will be password protected and only those who are involved in the research will have a password. These records are to be stored for a minimum of 5 years after the last clinical visit.

## Discussion

This study is designed to assess the effects of a pulsed dose of HF in the context of androgen deprivation as a mode of inducing DNA DSB that may potentiate the effects of radiation therapy for prostate cancer. This approach offers the potential for synergistic enhancement of standard radiation while utilizing an already-approved and clinically effective method of androgen deprivation, potentially exploiting a novel interaction and mechanism of action, namely type II topoisomerase TOP2B-mediated initiation of DNA DSBs. Given promising results in preclinical studies, we designed a two-phase study to assess the pharmacodynamic effects of this novel paradigm in inducing DSBs in addition to exploring the feasibility of experimental plasma- and urine-based methods of assessing therapeutic response. The two-phase design of the study allows for early verification of positive γH2Ax signal in an initial run-in cohort prior to continued enrollment of patients within the randomized portion of the trial, thereby minimizing the risk of unnecessary/unwarranted intervention in the full cohort of patients. We utilize a relatively novel method of biopsy under anesthesia during routine clinical care (brachytherapy), thereby reducing discomfort and enhancing patient ease of participation. The findings from this study will inform the development of a larger trial evaluating flutamide pulsed-dose sequencing in association with fractionated external beam RT and brachytherapy. In so doing, we hope to provide evidence for the development of a treatment regimen capable of optimizing the therapeutic index of conventional treatment.

## Trial status

At the time of manuscript submission, subject recruitment is ongoing and had recruited 18 patients. This trial began recruitment on May 10, 2019, and is expected to conclude on July 1, 2023. At the time of submission, run-in phase is completed with the null hypothesis rejected; randomization phase enrollment and data collection are ongoing.

## Abbreviations

PCa: Prostate cancer
AR: Androgen receptor
DHT: Dihydrotestosterone
DSBs: Double-strand breaks
TOP2B: DNA topoisomerase 2-beta
HF: Hydroxyflutamide
NAC: *N*-Acetyl-*L*-cysteine
ROS: Reactive oxygen species
IR: Ionizing radiation
RT: Radiotherapy
Gy: Gray
GHRH: Gonadotropin-release hormone
TURP: Transurethral resection of prostate
TRUS: Transrectal ultrasound
CTCAE: Common Terminology Criteria for Adverse Events
SAE: Serious adverse event
